# Heart of the matter

**DOI:** 10.1186/1749-8090-1-3

**Published:** 2006-03-03

**Authors:** David P Taggart

**Affiliations:** 1 Professor of Cardiovascular Surgery, University of Oxford, Oxford, UK

In the first issue of this new journal Denvir and colleagues [1] touch on a subject which is probably the single most important threat to cardiac surgery- the increasing tendency of some cardiologists to treat patients with severe coronary artery disease without a surgical opinion. Increasingly, the best interests of patients are no longer at the heart of the matter.

Throughout the developed world cardiac surgery faces increasing challenges from percutaneous intervention, currently with revascularisation, but potentially soon for valve replacement as well. The spectacular growth of PCI in the UK over the last five years is typical for many developed countries [2]. Since 1998 in the UK the ratio of PCI:CABG has increased from 1:1 to almost 3:1. This change in practice is likely to continue or even accelerate as the recent European Society of Cardiology [3] and the American Heart Association [4] guidelines recommend that PCI can be considered as the initial treatment strategy for most patients with multi-vessel disease. Furthermore these guidelines along with those of the British Cardiac Society [2], written almost exclusively by cardiologists (75 cardiologists and two surgeons), do not even recommend a surgical opinion, effectively dismissing the more effective option of CABG. In effect these guidelines promote abandonment of the multidisciplinary approach to multivessel ischaemic heart disease.

Most worrying, however, is that the European and American guidelines simply fly in the face of all the available evidence. The guidelines are based on a fundamentally flawed misunderstanding and/or misinterpretation of 15 randomised trials of PCI (five of which used stents) versus CABG. These trials reported that while CABG reduced the need for further intervention almost fourfold in patients with 'multi-vessel' disease there was no difference in survival between PCI and CABG. Yet this is simply not true for most patients with real multivessel ischaemic heart disease. The apparent similarity in survival was 'manufactured' by only including low risk patients in the trials. All the trial patients had normal ventricular function and around 70% had single or double vessel disease, a population in whom it had already been established that there was little prognostic benefit from surgery [5]. Around 95% of all the screened patients were excluded from these trials and in particular those who are known to benefit from surgery i.e. those with left main disease, severe and/or complex triple vessel disease, occluded vessels and with impaired ventricular function.

Accordingly, I previously wrote in the British medical Journal that these trials were in effect inherently biased against the prognostic benefits of surgery [6]. I also pointed out that the trials were subsequently presented in the medical and lay press in a disingenuous fashion. They were styled and titled as trials of patients with multi-vessel disease to imply that the trial patients had the typical pattern of triple vessel disease which is present in over 90% of CABG patients in the real world. Reporting of the trials and accompanying supportive editorials, almost exclusively written by cardiologists, disingenuously ignored or fleetingly mentioned their fundamental limitations.

Yet these trials have now been used to establish PCI as the default treatment for patients undergoing coronary revascularisation. They ignore the overwhelmingly strong evidence in favour of CABG in real clinical practice. In the New York registry of almost 60,000 risk matched patients in the "real world", those patients who received CABG rather than PCI had a highly significant absolute reduction in mortality of around 35% at three years and a seven fold reduction in the need for further intervention (5% vs 35%) [7]. Similar evidence in favour of CABG has also been reported from the Cleveland Clinic [8] and the effects are further magnified in diabetic patients [9]. An accompanying editorial to the New York Registry pointed out that the survival benefit of CABG is because as the bypass graft is placed to the mid coronary vessel, surgery deals not only with the culprit lesion, which can be of any complexity, but also has a protective effect against future culprit lesions.

As evidenced and inappropriately supported by the current ESC and AHA guidelines, an increasing proportion of current interventional cardiology practice appears to be driven by those either ignorant of or unwilling to acknowledge real facts. And evidence based medicine appears ineffectual against misleading trials and data backed and promoted by a multi-billion dollar industry.

So what is the best way to ensure that the patient at least has access to balanced advice regarding most effective treatment? I believe that this can only be achieved by the patient being advised by a multidisciplinary team, including a surgeon, who practice evidence based medicine [5]. The major European, Asian and American colleges of Cardiothoracic surgery should, individually and collectively, issue consensus guidelines to this effect. Ensuring that patients are appropriately and adequately informed ensures that they can make a rational decision about their treatment and sits well with medical and governmental recommendations to that effect. It might also provide some sense of check and balance to those cardiologists who, to the detriment of the patients, currently boast that they "do not refer patients for coronary artery bypass grafting".
